# Optimizing SH-SY5Y cell culture: exploring the beneficial effects of an alternative media supplement on cell proliferation and viability

**DOI:** 10.1038/s41598-024-55516-5

**Published:** 2024-02-27

**Authors:** Zeynep Bengisu Kaya, Valeria Santiago-Padilla, Melina Lim, Suelen L. Boschen, Pergin Atilla, Pamela J. McLean

**Affiliations:** 1 Department of Neuroscience, Mayo Clinic, 4500 San Pablo Road, Jacksonville, FL 32224 USA; 2 School of Medicine, University of Puerto Rico, San Juan, PR USA; 3 Department of Histology and Embryology, Hacettepe University Faculty of Medicine, Ankara, Turkey

**Keywords:** Biological techniques, Cell biology, Neuroscience

## Abstract

In the quest to unravel the mysteries of neurological diseases, comprehending the underlying mechanisms is supreme. The SH-SY5Y human neuroblastoma cell line serves as a crucial tool in this endeavor; however, the cells are known for its sensitivity and slow proliferation rates. Typically, this cell line is cultured with 10% Fetal Bovine Serum (FBS) supplement. Nu-Serum (NuS), a low-protein alternative to FBS, is promising to advance cell culture practices. Herein, we evaluated the substitution of NuS for FBS to test the hypothesis that an alternative serum supplement can aid and promote SH-SY5Y cell proliferation and differentiation. Our findings revealed that the NuS-supplemented group exhibited a notable increase in adhered cells compared to both the FBS and serum-free (SF) groups. Importantly, cell viability remained high in both sera treated groups, with the NuS-supplemented cells displaying significantly larger cell sizes compared to the SF-treated group. Furthermore, cell proliferation rates were higher in the NuS-treated group, and neuroblast-like morphology was observed earlier than FBS group. Notably, both FBS and NuS supported the differentiation of these cells into mature neurons. Our data supports NuS as an alternative for SH-SY5Y cell culture, with the potential to elevate the quality of research in the neuroscience field.

## Introduction

Neurological disease studies aim to unravel the complex interactions between genetic, molecular, and environmental factors that contribute to disease onset and progression by focusing on key cellular pathological processes, such as protein misfolding and aggregation, oxidative stress, inflammation, and impaired synaptic function^1,2^.

In vitro experiments, particularly those utilizing neuronal cell lines, play a vital role in advancing our understanding of neurodegenerative diseases as they provide insights into neuronal physiology, synaptic function, and neural network dynamics. However, culturing primary neurons is challenging and laborious because mature neurons do not divide, and the cell culture is not continuous. Considering these disadvantages, immortalized neuronal cell lines derived from neuronal tumors have become invaluable tools for mimicking the properties and behaviors of neurons found in the human brain^1,3^.

SH-SY5Y human neuroblastoma cells are one of the most widely used cellular models to study neurodegenerative diseases^4^. SH-SY5Y cells can proliferate continuously and as undifferentiated cells they present a neuroblast-like morphology and express immature neuronal markers. SH-SY5Y cells can be differentiated into neurons by the addition of retinoic acid (RA) and neurotrophins, become morphologically similar to primary neurons and losing their ability to proliferate and expressing neuron-specific markers^5^. Differentiated SH-SY5Y cells offer the advantage to study impaired dopamine metabolism and some other disease specific pathologies^6^. Even though SH-SY5Y cells offer many advantages, these cells are extremely sensitive and difficult to maintain, and proliferation tends to be slow, which makes their employment in routine lab practices challenging for researchers.

When culturing SH-SY5Y cells, specific methods and conditions are essential to maintain their viability and neuronal characteristics. Typical culture conditions for this cell line involve DMEM F12 supplemented with 10% FBS (Fetal Bovine Serum) and incubation in a humidified incubator with 5% CO_2_ at 37 °C^7^. FBS is the most common serum supplement used in cell culture media, however, it is not completely innocuous. FBS is derived from bovine fetuses and can pose risks for contamination due to its animal origin and represents obvious ethical concerns regarding animal welfare. Additionally, batch-to-batch variability can impact experimental reproducibility and reliability. As a result, there is a growing emphasis on finding alternative, animal-free culture media and serum substitutes to address these ethical concerns and promote more humane and sustainable practices as well as minimize the risk of animal protein contamination and enhance experimental consistency in cell culture^8^.

NuS (Nu-Serum) is a serum substitute developed as an alternative to animal-derived serum supplements like FBS. It is a defined low-animal-protein supplement, allowing for more consistency among batches and minimizing the risks of contamination^9^. NuS also addresses the ethical concerns related to animal welfare by reducing the reliance on animal-derived sera and providing sustainable and humane alternatives in cell culture practice.

In this study, we hypothesize that culturing SH-SY5Y cells with NuS will improve and promote cell proliferation, viability and differentiation when compared to the traditional culture methods. To test this hypothesis, cells were cultured in either DMEM F12 with 10% FBS, DMEM F12 with 10% NuS, or serum-free (SF) media conditions. Cell counts, cell size, and viability were evaluated by an automated cell counter and cell proliferation measurements were assessed by WST-1 assay. Cells were observed under brightfield microscope each day to assess morphology. Furthermore, we confirmed differentiation of undifferentiated SH-SY5Y cells into neuron-like cells in both FBS and NuS supplement conditions. Through immunofluorescent labeling, we morphologically compared the cellular development of FBS-treated cells and NuS-treated cells. Overall, our results support NuS as a potentially superior serum alternative for SH-SY5Y cell culture. NuS offers advantages of improved cell proliferation rates, viability, and morphology.

## Results

### Effect of NuS treatment on neuroblast-like morphology development and cell proliferation in undifferentiated SH-SY5Y cells

Undifferentiated SH-SY5Y cells exhibit neuroblast-like morphology, characterized by nonpolarized cell bodies and few truncated processes. These cells tend to grow in clusters, forming clumps of rounded cells on top of each other in the central region of a cell mass. At the periphery of the clusters, some cells begin to extend short neurites^5^.

**Figure 1:**
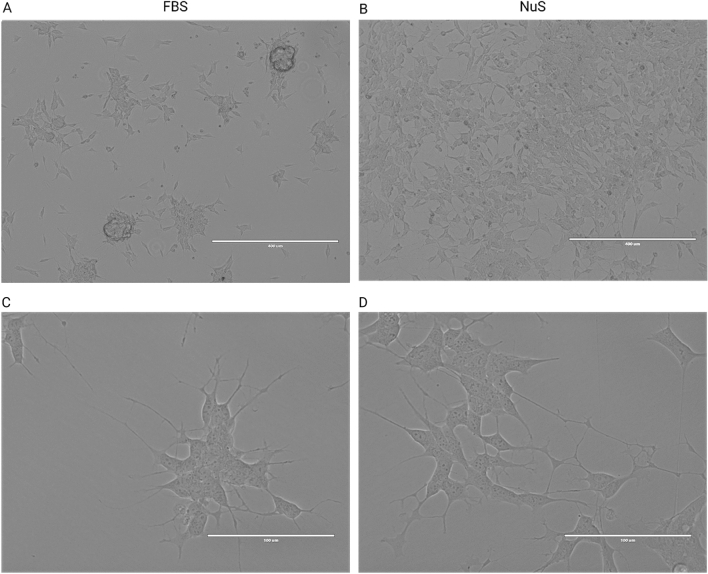
EVOS microscopy images of undifferentiated SH-SY5Y cells treated with FBS and NuS. (**A**) FBS-treated cells form clusters and grow on top of each other, resulting in densely populated clusters. (**B**) NuS-treated cells gradually spread and proliferate throughout the flasks by the end of the seven-day observation period. Scale bar = 400 μm. (**C**) FBS-treated cells displayed early-stage neuron-like features. (**D**) NuS-treated cells exhibited longer and better-developed cytoplasmic extensions, suggesting accelerated neuron-like morphology development. Scale bar = 100 μm.

In our study, we observed that SH-SY5Y cells, supplemented with both FBS and NuS, initially formed clusters after day two. SH-SY5Y cells treated with FBS continued to cluster on the plates and grow on top of each other (Fig. 1A), while NuS-treated cells initially formed clusters that then became more uniform throughout the culture flasks by the end of the seven-day observation period (Fig. 1B). High-magnification imaging revealed that FBS-treated cells displayed early-stages of neuron-like morphology, including a prominent nucleus, rounded or oval-shaped cell bodies with poorly developed, short, and thin neurite like cytoplasmic extensions (Fig. 1C). In comparison, the NuS-treated cells exhibited an elongated shape with longer and better-developed cytoplasmic extensions (Fig. 1D).

**Figure 2:**
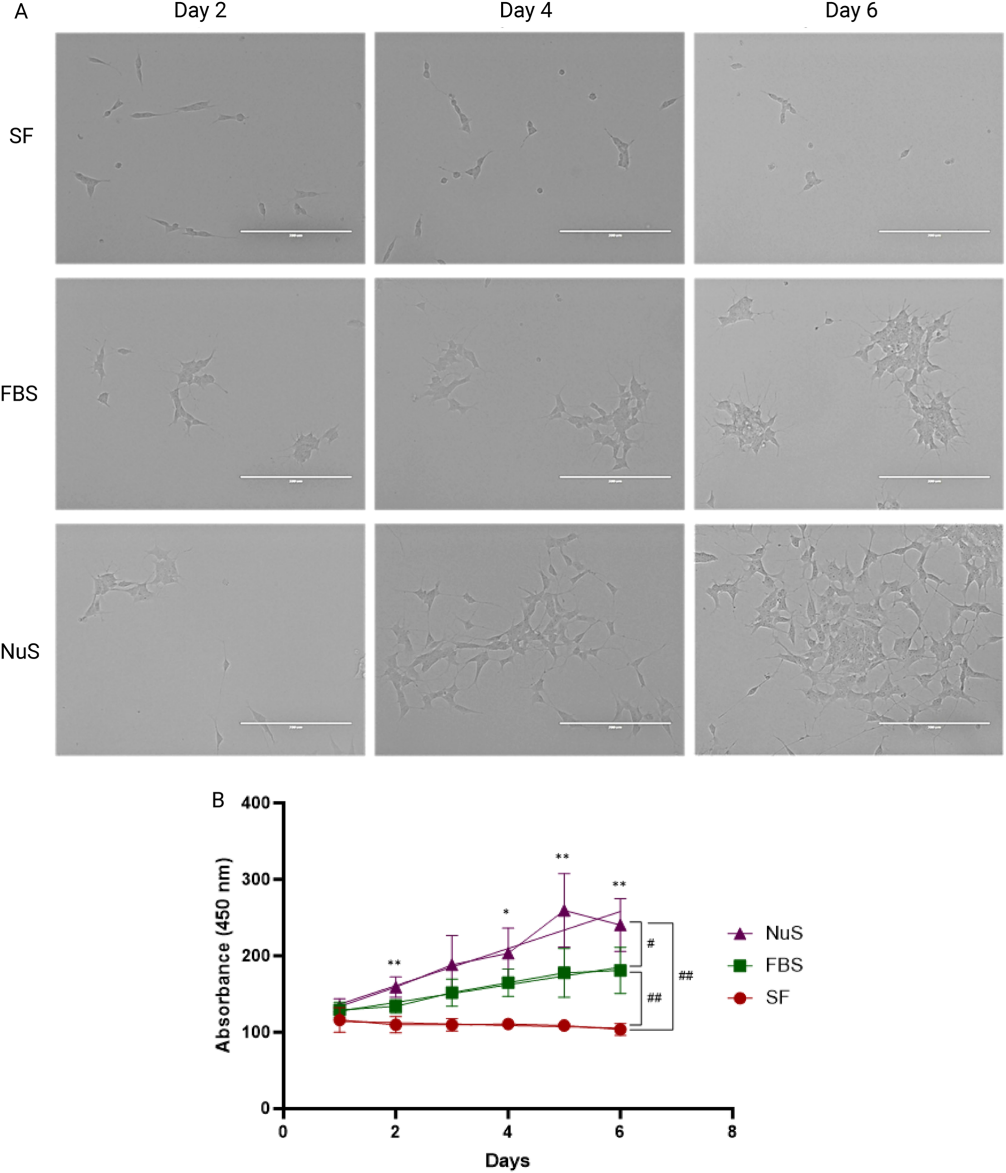
NuS treatment accelerates neuron-like development and proliferation in SH-SY5Y cells. (**A**) EVOS microscopy images of undifferentiated SH-SY5Y cells in SF group, FBS group, and NuS group on day 2, day 4, and day 6. Scale bar = 200 μm. (**B**) Cell proliferation quantitation (n = 8 biological replicates). Cell proliferation assays were conducted from day 1 through day 6 of the culture period. Data are analyzed with Two-way ANOVA followed by Tukey’s multiple comparison test and paired t-test and are represented as mean ± SEM. #*p* ≤ 0.05, ##*p* ≤ 0.01, *p ≤ 0.05, **p ≤ 0.01.

The morphological developments observed over the one-week period (Fig. 2A) were further supported by a time-course proliferation assay. To assess cell proliferation in growing cells, a WST-1 assay was conducted from day 1 through day 6 in SF, FBS, and NuS groups. The results demonstrated that NuS significantly accelerated cell proliferation compared to both SF and FBS conditions with the FBS-treated cells exhibiting significantly higher proliferation rates compared to the SF group (Fig. 2B). Furthermore, daily readouts revealed a consistent and significant promotion of cell proliferation in the presence of NuS supplementation (Fig. 2B). These findings highlight the effective role of NuS treatment in enhancing cell growth.

**Figure 3:**
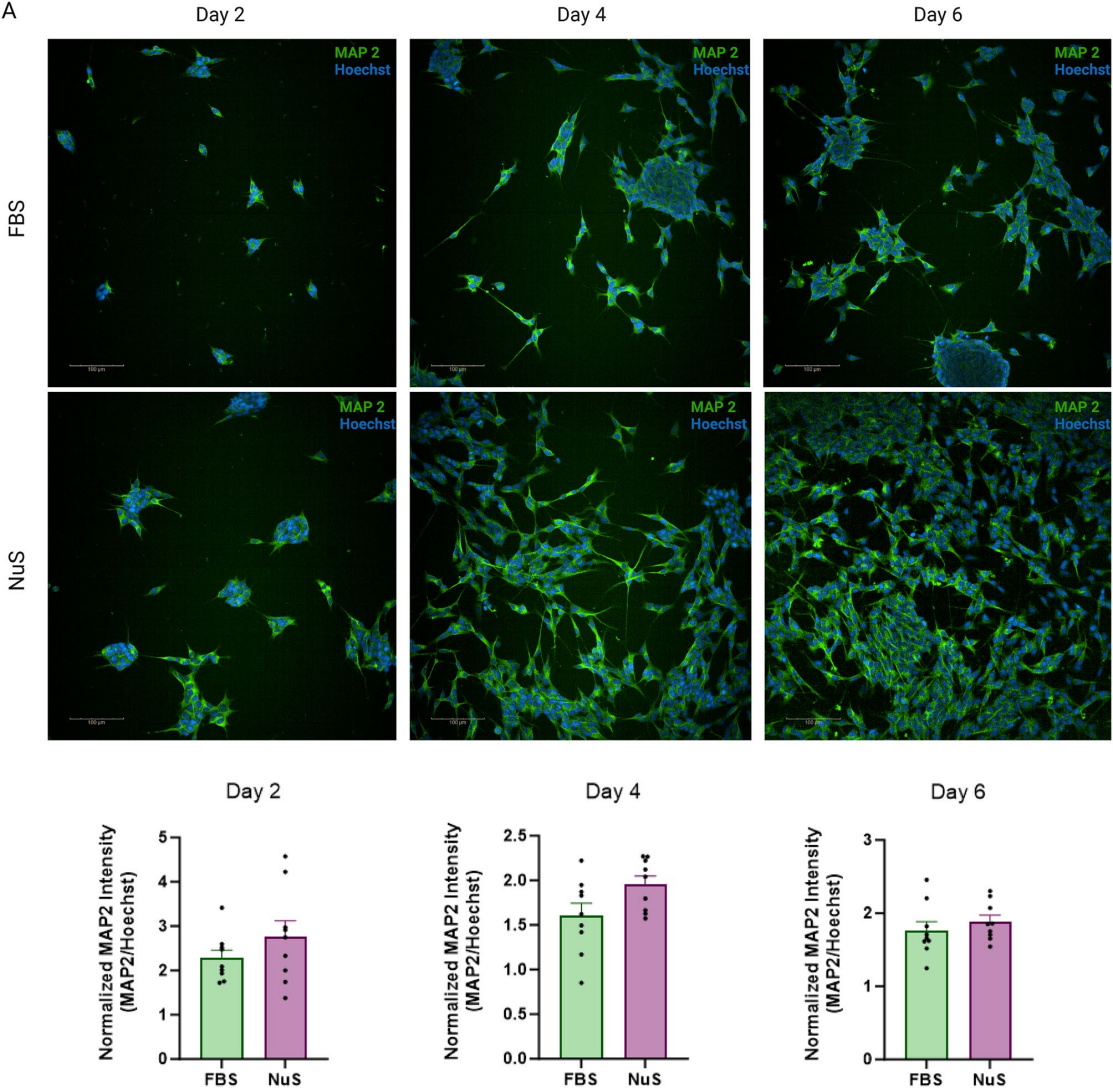

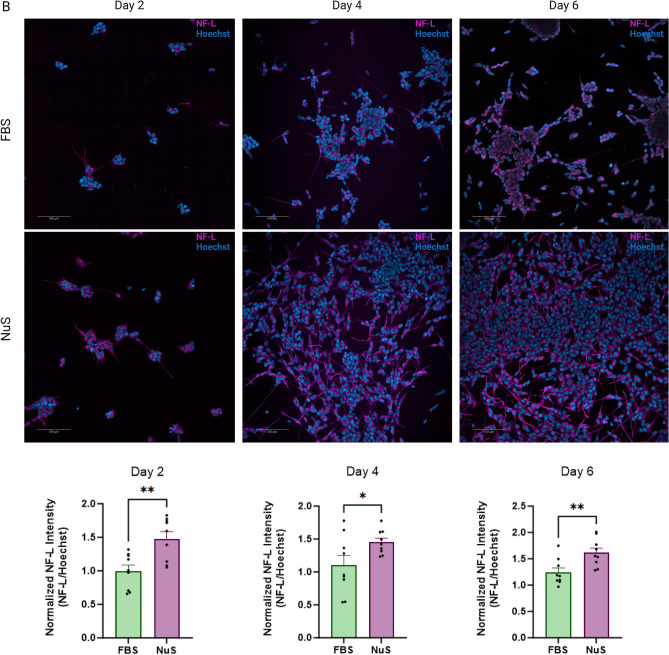
NuS treatment accelerates neuron-like morphology development in cells. Immunofluorescent images for MAP2 (**A**) and NF-L (**B**) of SH-SY5Y cells in FBS group and NuS group on day 2, day 4, and day 6. Nuclei in blue, MAP2 in green, NF-L in magenta. Scale bar = 100 μm. Bar graphs are representing the IF intensity quantification reflecting the MAP2/Hoechst (**A**) and NF-L/Hoechst (**B**) intensity, n = 9 areas. Data are analyzed with Unpaired t-test and are represented as mean ± SEM. *p ≤ 0.05, **p ≤ 0.01.

IF labeling supports these observations, demonstrating increased cell population in the NuS group compared to the FBS group. Additionally, the NuS-treated cells displayed better-developed perikarya and cytoplasmic extensions when labeled with neuron-specific cytoskeleton markers Microtubule-associated protein 2 (MAP2) (Fig. 3A) and Neurofilament Light (NF-L) (Fig. 3B). Additionally, quantification of IF images revealed a significant difference in development of cellular extensions and morphology in cells supplemented with NuS compared to FBS (Fig. 3). These findings suggest that NuS treatment accelerates neuron-like morphology development in undifferentiated SH-SY5Y cells.

### NuS treatment enhances cell concentration, viability, and cell size compared to FBS and SF treatments

**Figure 4:**
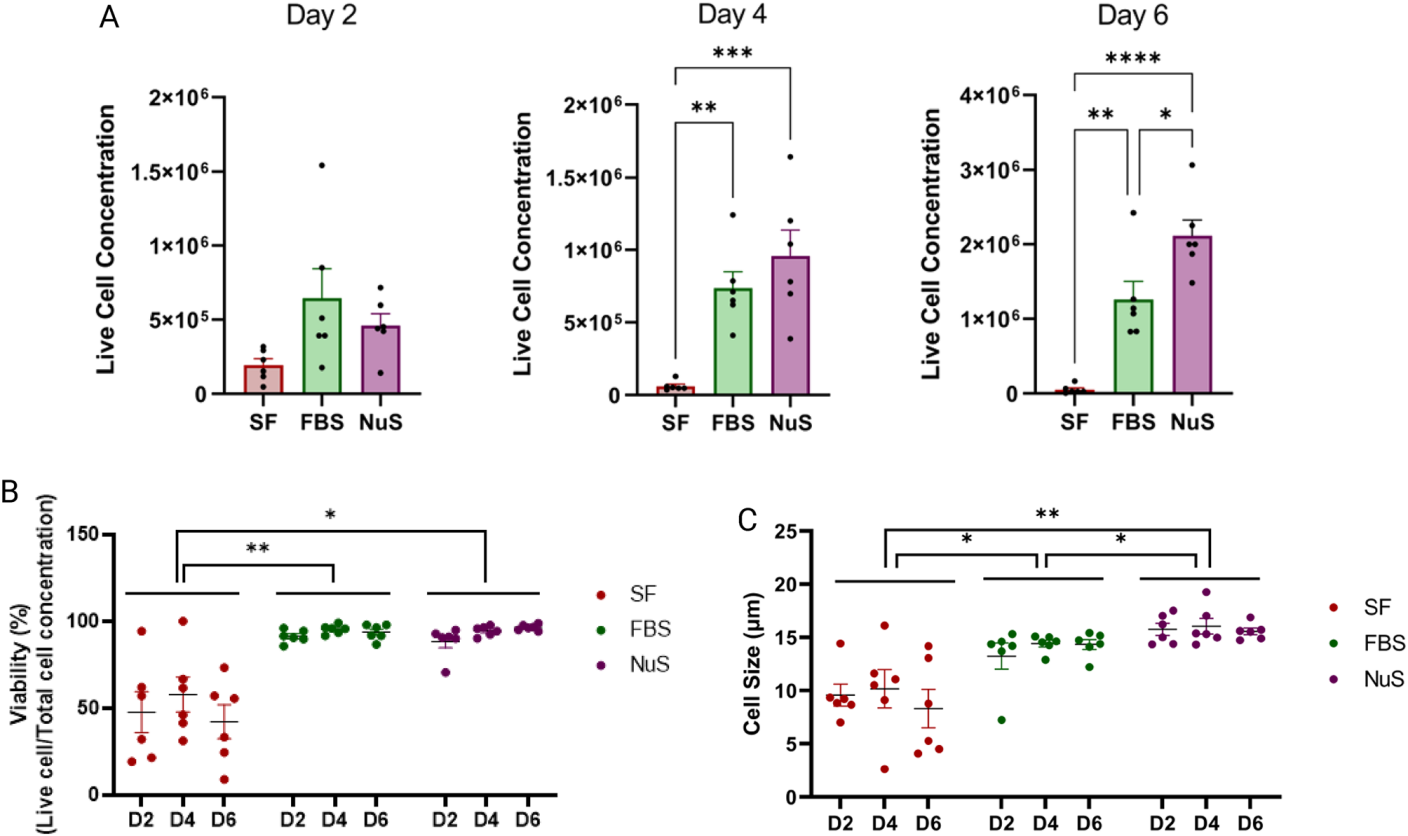
NuS treatment enhances cell number, viability, and size compared to FBS and SF treatments. (**A**) Cell counts (**B**), viability, and (**C**) size were measured on days 2, 4, and 6 (n = 6 biological replicates). (**p ≤ 0.01, ***p ≤ 0.001, ****p ≤ 0.0001). Data are analyzed with Two-way ANOVA followed by Tukey’s multiple comparison test and unpaired t-test and are represented as mean ± SEM. *p ≤ 0.05, **p ≤ 0.01, ***p ≤ 0.001, ****p ≤ 0.0001.

In this study, cell counting, cell size, and viability were evaluated on days 2, 4, and 6 using an automated cell counter. The results demonstrated that NuS treatment significantly influenced cell concentration, as evidenced by significantly higher total (data not shown) and live cell numbers compared to the FBS and SF groups. Additionally, the FBS group had significantly higher cell numbers than the SF group (Fig. 4A). Cell viability (live cell concentration/total cell concentration) analysis revealed that both NuS and FBS groups exhibited significantly higher viability than the SF group, although they were not different from each other (Fig. 4B). Cell size measurements indicated that cells in both FBS and NuS groups were larger compared to the SF group (Fig. 4C). Remarkably, cells in the NuS group were significantly larger than the FBS group overall (Fig. 4C). These findings suggest that NuS treatment promotes favorable cellular outcomes for undifferentiated SH cells with improved growth and health.

### SH-SY5Y cells supplemented with NuS can be differentiated

**Figure 5:**
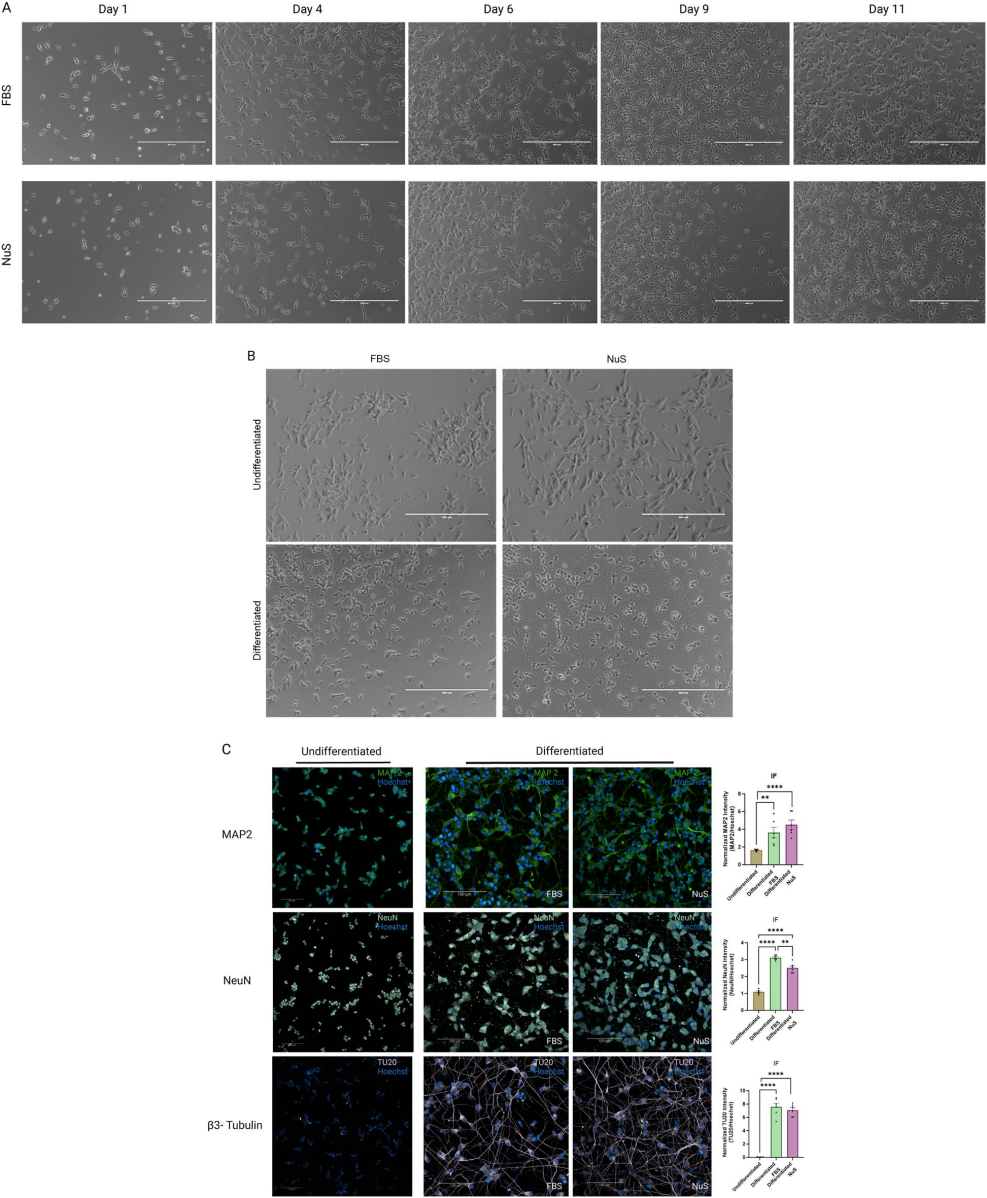
SH-SY5Y cells can be differentiated using NuS supplement. (**A**) EVOS microscopy images of differentiating SH-SY5Y cells in FBS group and NuS group on day 1, day 4, day 6, day 9 and day 11. Scale bar = 400 μm. (**B**) EVOS microscopy images of undifferentiated and differentiated (on day 11) SH-SY5Y cells in FBS and NuS groups. Scale bar = 400 μm. (**C**) Immunofluorescent images for MAP2, NeuN and β3-Tubulin neuronal markers on undifferentiated and differentiated SH-SY5Y cells. Nuclei in blue, MAP2 in green, NeuN in light green, β3-Tubulin in violet. Scale bar = 100 μm. Bar graphs are representing the IF intensity quantification reflecting the MAP2/Hoechst, NeuN/Hoechst and TU20/Hoechst intensity, n = 6 areas. Data are analyzed with Unpaired *t*-test and are represented as mean ± SEM. **p ≤ 0.01, ****p ≤ 0.0001.

The differentiation of SH-SY5Y cells was successfully induced in the presence of FBS or NuS supplements. A qualitative analysis of the two conditions did not reveal significant morphological differences in differentiated cells. Undifferentiated SH-SY5Y cells exhibited a neuroblast-like morphology and continued proliferating as mentioned above. The addition of RA led to morphological changes and the acquisition of a more mature neuronal-like phenotype. Following an 11-day differentiation period, cells displayed a polarized cell body structure accompanied by extended, prominent, and branching neurites (Fig. 5A). Additionally, differentiated cells appeared to extend their neurites towards neighboring clusters, presumably to establish synaptic connections (Fig. 5B).

Differentiated cells are expected to express mature neuronal markers, such as MAP2, NeuN, and beta III Tubulin (β3-Tubulin)^7,10,11^. We labelled undifferentiated and differentiated cells to confirm maturation of SH-SY5Y cells into neurons. Notably, both undifferentiated and differentiated cells expressed MAP2 and NeuN markers. However, differentiated cells exhibited a distinct profile of a neuron, characterized by robust expression of neuronal markers aligned with developed neuronal morphology and neurite outgrowth (Fig. 5C). Conversely, the expression of β3-Tubulin, a marker associated with mature neurons, was exclusively detected in the differentiated cells, aligning with the well-developed, mature neuron-like cytoskeletal structure of the cells (Fig. 5C). Quantification of IF images revealed a significantly higher expression of mature neuronal markers in differentiated cells supplemented with either FBS or NuS compared to the undifferentiated cells. These results demonstrate the successful differentiation of SH-SY5Y cells regardless of FBS or NuS supplements, observed as morphological changes and a mature neuronal phenotype supported by the expression of mature neuronal markers.

## Discussion

Neurological diseases are complex and debilitating disorders that pose significant challenges to both researchers and patients. In vitro experiments, particularly those utilizing neuronal cell lines, have proven to be invaluable tools in unraveling the underlying mechanisms of neurodegenerative diseases^12^.

NuS was developed as an alternative to animal-derived serum supplements to provide the necessary growth factors and nutrients for cell culture and offers advantages including a defined and low-animal-protein composition, consistent batch-to-batch performance, enhanced experimental reproducibility, and reduced risk of contamination with animal pathogens. Moreover, NuS addresses ethical concerns related to animal welfare, making it a more humane and sustainable option for cell culture. NuS finds utility in a wide range of research areas such as neuroscience studies^13,14^, cancer research^15,16^, regenerative medicine and tissue engineering studies^17^, immunology^18^, cell signaling^19^, and drug discovery^20,21^ research.

FBS is derived from bovine fetuses and contains various growth factors, hormones, and other nutrients that support cell growth^8,22^. Due to its animal origin, there is a risk of contamination with pathogens, including bacteria, viruses, prions, and mycoplasma. This can pose a significant concern for both cell culture experiments and downstream applications, as well as lab staff. Furthermore, FBS is a complex mixture with undefined composition and batch-to-batch variability. The lack of standardized quality control measures and variations in protein concentrations can impact experimental reproducibility and reliability^22,23^. Additionally, the use of animal-derived serum supplements, such as FBS, raises ethical concerns. These supplements are obtained from the blood of unborn animals, often through procedures that involve slaughtering pregnant cows^8,23^. This raises ethical questions about animal welfare, sustainability, and the use of animal-derived products in scientific research. As a result, there is a growing emphasis on finding alternative, animal-free culture media and serum substitutes to address these ethical concerns and promote more humane and sustainable practices as well as to minimize the risk of animal protein contamination and enhance experimental consistency in cell culture^8^.

In this study, we investigated the effects of NuS supplement on undifferentiated SH-SY5Y cells compared to SF media and traditional culture methods using FBS supplement. By exploring the potential benefits of NuS supplementation, we sought to overcome the limitations and challenges associated with SH-SY5Y cell line, offering researchers a promising and reliable serum substitute.

We successfully demonstrated that NuS treatment significantly enhances cell concentration, viability, and cell size compared to FBS and SF treatments. Moreover, cells treated with NuS exhibit a more neuron-like morphology characterized by elongated shapes and well-developed cytoplasmic extensions compared to FBS-treated cells. NuS treatment significantly accelerated cell proliferation compared to SF and FBS conditions and supported differentiation of SH-SY5Y cells into mature neurons with comparable efficacy as the traditional FBS supplementation method. Although the extensive branching seen in fully differentiated neurons was not observed in either group, the elongated shape and improved neurite-like projections in the NuS-treated cells indicate their potential to differentiate into more mature neuronal phenotypes. The specific mechanisms underlying enhanced effectiveness of NuS may involve several factors. FBS is known for its batch-to-batch variability, which can impact experimental reproducibility. NuS, with standardized formulation, potentially offers more reliable and reproducible results within cell culture practices. FBS, due to its animal origin, can introduce immunogenic factors and contaminants that may affect cell behavior. In contrast, NuS, containing low-animal-protein, may provide a more supportive environment for cell growth.

The widespread use of SH-SY5Y cells in neuroscience research is a testament to their utility and relevance in advancing our understanding of neurodegenerative diseases^4^. By providing a reliable and reproducible model, SH-SY5Y cells have contributed significantly to the discovery of potential therapeutic targets and the development of novel treatment approaches^24^. Our data support NuS as a promising supplement for supporting the growth and health of SH-SY5Y cells in cell culture. These findings hold implications for neurological disease studies and highlight the importance of serum substitutes in cell culture practice.

In conclusion, the results of this study provide insights into the effects of NuS treatment on cell proliferation, viability, and morphology and thus contribute to the field of neuroscience research. Our data support NuS as a promising supplement for supporting the growth and health of SH-SY5Y cells in culture. These findings hold implications for neurological disease studies and highlight the importance of serum substitutes in cell culture practice. The findings open avenues for further investigations into the mechanisms through which NuS treatment exerts its beneficial effects on cell growth and differentiation. Furthermore, the potential of NuS extends beyond neurobiology, making it a valuable tool in diverse research fields such as cancer, regenerative medicine, and drug discovery. The significance of this study lies in the potential improvements it offers in the quality and reliability of cell culture experiments, ultimately contributing to the advancement of neurobiology and neurodegenerative disease research.

## Methods

### Cell culture

SH-SY5Y cells (ATCC, CRL-2266) were maintained at 37 °C in a 5% CO_2_ humidified incubator. Prior to the experiments cells were cultured in DMEM F12 (11320-033, ThermoFisher) + 10% FBS (10,437,028, ThermoFisher) and DMEM F12 + 10% NuS (355504, Corning) conditions. Cells were tested for mycoplasma contamination at regular intervals. For further experiments, cells were divided into three groups. These cells were plated with SF media (SF group), 10% FBS-containing media (FBS group), and 10% NuS-containing media (NuS group).

### Cell counting, cell size, viability, and morphology analysis

Cell morphology was observed using an EVOS phase-contrast microscope each day (Invitrogen, ThermoFisher Scientific, USA). Cell counting, cell size, and viability were assessed using an automated cell counter, Countess II (Invitrogen, ThermoFisher Scientific, USA), on days 2, 4, and 6 of the culturing periods. The Countess II is a reliable instrument that utilizes image-based analysis to accurately determine cell numbers and measure cell size. SH-SY5Y cells were plated in a 6-well plate (3516, Corning Costar) with 300,000 cells per ml in SF, 10% FBS, and 10% NuS media. To perform the analysis, cells were harvested by applying trypsin (Trypsin–EDTA (0.25%), 25200056, Gibco) on the day of measurements. After centrifugation, cell pellets were suspended in 1 ml media to ensure an appropriate cell density for counting. Ten microliters from the diluted cell suspension was mixed with 10 µl Trypan Blue Stain (0.4%) (T10282, Invitrogen, Thermo Fisher Scientific) then loaded onto the Countess II, and the instrument automatically captured images of the cells and analyzed the data. The cell counting feature provided quantitative information on cell numbers (total and live cell concentration per ml), while the cell size analysis allowed for the determination of average cell size. Additionally, the viability of the cells was assessed enabling the calculation of cell viability based on the ratio of live to total cells. Cell counting, size and viability experiments were performed in six replicates including two repetitions for each group in each set.

### Cell proliferation assay

Cell proliferation was determined using WST-1 (ab155902, abcam) assay according to manufacturer instructions. Briefly, SH-SY5Y cells were plated in a 96-well plate (655,098, Fisher) with 10,000 cells per well in 100 µl of SF, 10% FBS, and 10% NuS media. Starting from the following day, each day 10 µl/well of WST-1 reagent was added to wells for six days. The plate was incubated at 37 °C for 4 h before the WST-1 absorbance was read at OD 450 nm by EnVision microplate reader (EnVision, PerkinElmer). Cell proliferation assay was performed in eight replicates including three repetitions for each group in each set.

### SH-SY5Y cell differentiation

Undifferentiated SH-SY5Y cells were maintained in a basic growth medium consisting of DMEM F12 with Glutamax (10565018, Gibco Life Technologies). The media was supplemented with either 10% heat-inactivated FBS (hiFBS) (10437028, Gibco Life Technologies) or 10% NuS (355504, Corning), along with 1% penicillin/streptomycin (15140122, Gibco Life Technologies).

For differentiation protocol^7,11^, cells were plated in matrigel coated (354277, Corning) black 24 well plates (P24-1.5P, Cellvis) in 30,000 per well density on Day 0. The following day (Day 1), the medium was changed to Stage I media, composed of DMEM F12 with Glutamax supplemented with 2.5% hiFBS or 2.5% NuS, 1% penicillin/streptomycin, and 10 μM RA (R2625, Sigma Aldrich). A media change with Stage I media was repeated on Day 4. On Day 6, the medium was switched to Stage II media which consisted of Neurobasal media (21103049, Gibco Life Technologies) supplemented with 50 ng/mL brain-derived neurotrophic factor (BDNF) (B-250, Alomone Labs), 20 mM potassium chloride (KCl), B-27 supplement (17504044, Gibco Life Technologies), 0.2 mM dibutyryl cyclic AMP (db-cAMP) (#73882, StemCell Technologies), 2 mM Glutamax (35050-061, Gibco Life Technologies), and 1% penicillin/streptomycin (15140122, Gibco Life Technologies). A media change with Stage II medium was repeated on Day 9. On Day 11, the differentiated SH-SY5Y cells were considered ready for immediate use or subsequent experiments. The immunofluorescent protocol was initiated on Day 11 of differentiation. During the differentiation process, cells were observed and imaged by EVOS phase-contrast microscope each day (Invitrogen, ThermoFisher Scientific, USA).

### Immunofluorescence

In order to observe proliferation and morphological development, immunofluorescent (IF) labeling was performed. Cells were plated in matrigel coated (354277, Corning) black 24 well plates (P24-1.5P, Cellvis) in 50,000 cells per well density. Plates were designated to IF imaging on day 2, 4, and 6 after plating. Plates imaged on day 4 and day 6 had the media changed every two-days until imaging. On the day of IF labeling cells were washed with phosphate-buffered saline (PBS), fixed with 4% paraformaldehyde (PFA) for 15 min, permeabilized with 0.03% Triton-X for 15 min, and blocked with 2% normal goat serum (NGS) for 1 h at room temperature (RT). Cells were incubated overnight at 4 °C with anti-MAP2 (ab5392, abcam), anti-NF-L (#2837, Cell Signaling) primary antibodies prepared in PBS containing 1% BSA. The next day, cells were washed and treated with Alexa Fluor 488 (Invitrogen, ThermoFisher) secondary antibody for 1 h at RT. Next, cells were washed with PBS and nuclei were stained with Hoechst 33342 (H 3570, Invitrogen). Finally, cells were visualized using an Operetta CLS high content analysis system (Perkin Elmer) and 9 random areas for each group at days 2, 4, and 6 were analyzed. Fluorescence intensities of MAP2 and NF-L were evaluated, and intensities were normalized to the intensity of Hoechst with Image J^25^.

Undifferentiated and differentiated SH-SY5Y cells were labeled with neuron-specific markers to observe the differentiation. At the end of differentiation protocol IF was performed as described above. Cells were incubated overnight at 4 °C with anti-MAP2 (ab5392, abcam), anti-NeuN (ab 104225, abcam), and anti-β3-Tubulin (TU20) (#4466, Cell Signaling) primary antibodies and the next day, cells were washed and treated with Alexa Fluor 488 (Invitrogen, ThermoFisher) secondary antibody. Next, cells were washed with PBS and nuclei were stained with Hoechst 33342 (H 3570, Invitrogen). Finally, cells were visualized using an Operetta CLS high content analysis system (Perkin Elmer) and 10 random areas for each group were analyzed. Fluorescence intensities of MAP2, NeuN and β3-Tubulin were evaluated, and intensities were normalized to the intensity of Hoechst with Image J^25^ in 6 of the analyzed areas.

### Statistical analysis

All data were analyzed using Graph Pad Prism 9.0.0 software (San Diego, CA). Firstly, the normality of the data was assessed using the Kolmogorov–Smirnov test. Next, to compare the significance of differences between groups, one-way analysis of variance (ANOVA) was conducted followed by Tukey’s post-hoc test for multiple comparison and Unpaired t-test was used for additional pairwise comparisons. Results presented as mean ± standard error of the mean (S.E.M.) within 95% confidence interval, and *p* < 0.05 values were considered statistically significant (*p ≤ 0.05, **p ≤ 0.01, ***p ≤ 0.001, ****p ≤ 0.0001).

## Data Availability

All data generated or analyzed during this study are included in this published article.
